# Apparent latent structure within the UK Biobank sample has implications for epidemiological analysis

**DOI:** 10.1038/s41467-018-08219-1

**Published:** 2019-01-18

**Authors:** Simon Haworth, Ruth Mitchell, Laura Corbin, Kaitlin H. Wade, Tom Dudding, Ashley Budu-Aggrey, David Carslake, Gibran Hemani, Lavinia Paternoster, George Davey Smith, Neil Davies, Daniel J. Lawson, Nicholas J. Timpson

**Affiliations:** 1 Medical Research Council Integrative Epidemiology Unit, Department of Population Health Sciences, Bristol Medical School, University of Bristol, Oakfield House, Oakfield Grove, Bristol, BS8 2BN UK; 2 Avon Longitudinal Study of Parents and Children, Oakfield House, Oakfield Grove, Bristol, BS8 2BN UK

## Abstract

Large studies use genotype data to discover genetic contributions to complex traits and infer relationships between those traits. Co-incident geographical variation in genotypes and health traits can bias these analyses. Here we show that single genetic variants and genetic scores composed of multiple variants are associated with birth location within UK Biobank and that geographic structure in genotype data cannot be accounted for using routine adjustment for study centre and principal components derived from genotype data. We find that major health outcomes appear geographically structured and that coincident structure in health outcomes and genotype data can yield biased associations. Understanding and accounting for this phenomenon will be important when making inference from genotype data in large studies.

## Abstract (web summary)

Population structure can bias the results of genetic and epidemiological analysis. Here, Haworth et al. report that fine-scale structure is detectable in apparently homogeneous samples such as ALSPAC when measured very precisely, and remains detectable in UK Biobank despite conventional approaches to account for it.

## Introduction

Genotype data are used to answer an increasing number of research questions through an increasing number of methods. Latent structure within genetic data was originally identified as a problem in candidate gene association studies, where population stratification was “probably the most often cited reason for non-replication of genetic association results”^1^. With the advent of data at genome-wide scale, it became possible to parameterise the impact of latent structure on genetic association results using principal components (PCs)^2^ derived from genotype data among other approaches^3^. These methods, in conjunction with stringent genome-wide significance thresholds^4^ and the requirement for replication in an independent population, led to substantial improvement in replicability of genetic association studies.

In other contexts, detecting and accounting for latent structure remains problematic. Determining heritability of complex traits is challenging^5,6^ because population structure and polygenic traits both impart genome-wide signatures which may be truly (rather than spuriously) related. For example, genetic height^7^ varies amongst historical populations from which modern populations represent different mixtures. Stratification may be part of the explanation for different signatures of selection for height in UK Biobank compared to the GIANT consortium^8,9^. Analysis of rare genetic variation is another challenge, as the latent structure within this subset of genetic data may not reflect the latent structure of common variants used to generate PCs^10^.

An entirely different context for using genetic data is in epidemiological analyses which have developed substantially with the availability of reliable genetic association results from published sources and very large collections of genetic and phenotypic data such as UK Biobank^11^. A good example of this is Mendelian randomisation, which aims to escape confounding in associations by using genetic variation to proxy risk factors of interest^12^. Recent literature has focussed on maximising the use of the current wave of genetic association evidence and accounting for undesirable pleiotropic effects of single variants^13^. This activity has largely assumed that structure is addressed during the discovery of associated genetic variants, an assumption which now warrants closer examination. If present, latent structure within datasets used to perform epidemiological analyses would violate the requirement that genetic instrumental variables are not related to potentially confounding features^14^ and may result in biased epidemiological inference.

Here, we use geographical information in conjunction with genetic data to investigate latent structure (of unknown cause) in two population-based cohorts in the United Kingdom. We show that single genetic variants and polygenic scores incorporating multiple variants are associated with birth location in data from UK Biobank. Given regional differences in many health outcomes, this observed structure provides a source of covariance between genotypes and health outcomes which can bias epidemiological inference from genetic data. Understanding and accounting for this phenomenon will be important when making inference from genotype data in large studies.

## Results

### Alignment of educational attainment with ancestry

**Fig. 1:**
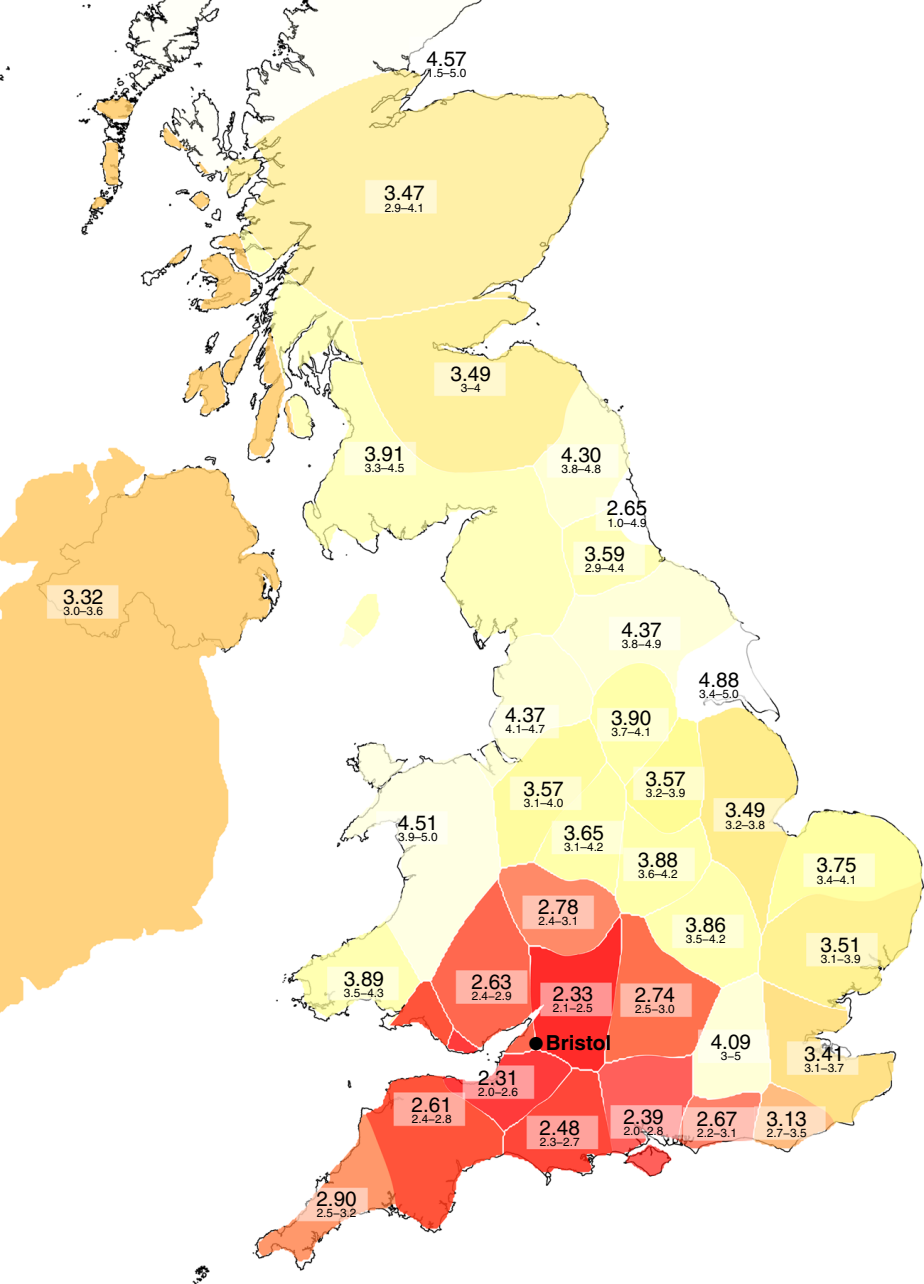
Within-UK ancestry predicts migration that confounds education: estimated educational attainment of the United Kingdom, when seen only through the ALSPAC cohort based in Bristol. Scores are 1: vocational, 2: CSEs, 3: O-levels, 4: A-levels, 5: degree. CSE Certificate of Secondary Education. The predicted mean education for each region is given, along with 95% confidence intervals estimated by bootstrap resampling of individuals. Each region is coloured by predicted mean education, where predicted mean = 2 is shaded in red and predicted mean = 5 is shaded in white. See Methods for details. ALSPAC Avon Longitudinal Study of Parents and Children

We examined whether there is previously under-appreciated structure in a well-understood ethnically and geographically homogenous resource using the the Avon Longitudinal Study of Parents and Children (ALSPAC)^15,16^ as an exemplar. We studied 7739 mothers who were recruited during pregnancy in the Bristol area (South West UK) in the early 1990s. We undertook chromosome painting^17^ to describe fine-scale relatedness between each mother and each of the regions of the Peopling of the British Isles (PoBI) project^18^, acting as an external source of geographical information. We summarised each mother’s ancestral lineage as a mixture of the PoBI regions, allowing us to estimate the educational attainment that those regions would have were the ALSPAC mothers’ education levels explained by this variation. In doing this a pattern for lower educational attainment in lineages originating from the regions immediately surrounding Bristol (Fig. 1) and higher educational attainment in more geographically distant lineages was observed. The patterns of educational attainment within the United Kingdom predicted by the ALSPAC sample are strikingly different from patterns of educational attainment observed in national surveys^19^. Distant lineages are likely only represented in ALSPAC by individuals or families who had migrated, and we anticipate that the educational attainment of people who migrate for economic reasons differs from people who do not. Educational attainment is therefore aligned to subtle genetic differences even in this apparently geographically and ethnically homogenous population and this is co-incident with axes of ancestry.

### Alignment of common genetic variants with geography

The structure in ALSPAC was detected here using a chromosome painting method, which is highly sensitive to ancestry. With greater power, it is entirely possible the same phenomena may become detectable in more routine analytical procedures for gene discovery or epidemiological analysis. We therefore turned to UK Biobank, an exceptional resource containing a catalogue of health, disease and genotype data of almost half a million participants^11,20^. Conceptually, UK Biobank is analogous to a super-imposition of multiple ALSPACs, each of which recruited participants living near a study assessment centre. This design not only gives UK Biobank the capacity to represent a broad spectrum of UK ancestry and structure, but also means that the study is sensitive to important sampling phenomena including self-selection. The hurdles of location and attendance (less than 6% of individuals contacted by UK Biobank chose to participate^21^) are likely to influence the nature of the resultant participant collection and are related to behaviours with heritable contributions^22^. This may create collider biases^23,24^ which have the ability to induce association between otherwise independent variables.

We examined whether there is geographic structure in the genetic data of UK Biobank using within-study geographical information by performing genome-wide association studies (GWASs) for birth location in PLINK^25^. The outcomes were North/South and East/West axes of birth location, both measured on a metre grid scale from an origin South West of the United Kingdom. Analysis of genetic data was performed within individuals of white British ancestry with non-missing data on birth location (*n* = 321,439). GWAS for birth location identified that single variants are associated with geography within UK Biobank. An unadjusted model produced distorted and inflated plots with evidence for association at variants across the autosome. After adjustment for genotyping array, 40 PCs and a factor variable representing UK Biobank assessment centre single variants remained associated with birth location (Supplementary Fig. 1).

### Alignment of polygenic scores with geography

**Fig. 2:**
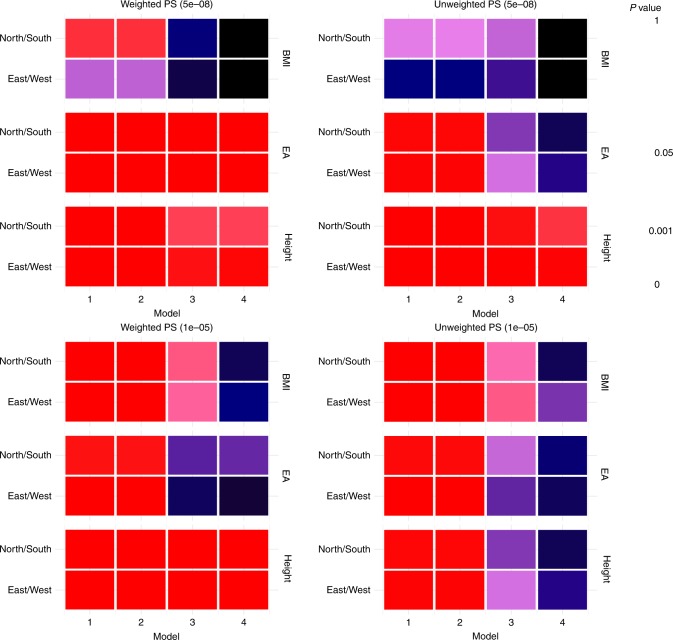
The relationship between polygenic scores (PS; right-hand label) and geographical terms (left-hand label) within the UK Biobank sample. Tiles are shaded by *p* value testing the null hypothesis of no association between PS and geographical term, where *p* = 0 is shaded in black and *p* = 2e−16 is shaded in red. Statistical adjustment was performed as follows: model 1: no adjustment; model 2: adjustment for genotyping array only; model 3: adjustment for genotyping array, 10 principal components (PCs) and study participation centre; model 4: adjustment for genotyping array, 40 PCs and study participation centre

**Fig. 3:**
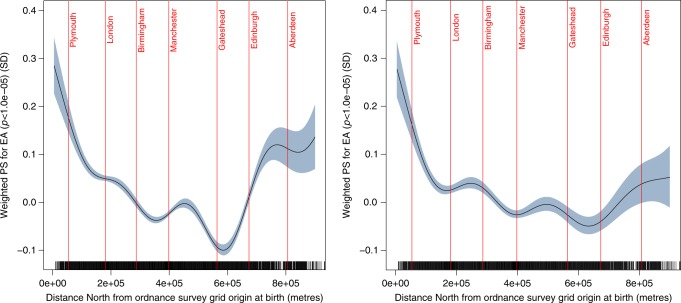
Fitted spline regression plots showing the non-linear distribution of polygenic scores (PS) for educational attainment (weighted version, including variants with *p* < 1.0e−05) in unadjusted model (left) and model after adjustment for 40 principal components and study centre (right). The centre of major population centres is marked for reference. The shaded area represents 95% confidence intervals

Rather than using single genetic variants, empirical epidemiological analyses often use polygenic scores (PS)^26,27^. As exemplars, we took genetic variants and weightings associated with educational attainment, height and body mass index (BMI) from published genome-wide meta-analyses^28–30^ excluding UK Biobank. Using an approach that is widespread in applied analyses, we used these externally derived variants and weightings in conjunction with the UK Biobank genetic data to create polygenic scores for the three traits. Aiming to understand the properties of these polygenic scores under a range of analytical contexts, we created both weighted and unweighted PS at a strict and more liberal threshold of association in the discovery sample (*p* < 5e−08 and *p* < 1e−05 respectively). We used general additive models^31^ in the ”mgcv” package (version 1.8)^32^ within R (version 3.3.1)^33^ to test for non-linear relationships between PS and geographical terms. All PS tested were associated with birth location in an unadjusted model and a model that adjusted only for genotyping array. These associations attenuated but were not extinguished in models incorporating adjustment for 40 PCs and study centre, especially for educational attainment and birth location on the North/South axis, where statistical adjustment had little impact on the fitted geographical distribution of the PS (Figs. 2 and  3 and Table 1). There is some irregularity in the pattern of geographical association when comparing the characteristics of weighted versus unweighted PS for the same trait or when comparing strictly defined versus liberally defined PS for the same trait, suggesting that the characteristics of these PS are sensitive to changes in composition. Sensitivity analyses using a PS for BMI trained in published data from Biobank Japan^34^ yielded similar findings to the PS for BMI trained in GIANT (Supplementary Table 1).

**Table. 1:** Relationship between PS and birth location within UK Biobank

		*P* value for association between PS and geographical term							
	Axis	Model 1	Model 2	Model 3	Model 4	Model 1	Model 2	Model 3	Model 4
*P* (5.0e−08)		BMI (GIANT)							
	N/S	9.7e−7	9.9e−7	0.063	0.40	0.0013	0.0012	0.0032	0.58
	E/W	0.0036	0.0035	0.24	0.93	0.053	0.054	0.032	0.47
		EA (SSGAC)							
	N/S	2e−16	<2e−16	6.4e−6	6.7e−6	<2e−16	<2e−16	1.3e−9	1.6e-6
	E/W	<2e−16	<2e−16	1.5e−9	6.0e−11	<2e−16	<2e−16	7.5e−14	1.3e-11
		Height (GIANT)							
	N/S	<2e−16	<2e−16	1.3e−5	0.14	<2e−16	<2e−16	4.6e−06	0.13
	E/W	<2e−16	<2e−16	2.1e−4	0.095	<2e−16	<2e−16	3.4e−05	0.046
*P* (1.0e−05)		BMI (GIANT)							
	N/S	2.4e−9	2.5e−09	0.023	0.019	2.4e−10	2.6e−10	0.0029	0.074
	E/W	1.4e−13	1.7e−13	0.134	0.34	<2e−16	<2e−16	0.020	0.14
		EA (SSGAC)							
	N/S	<2e−16	<2e−16	<2e−16	<2e−16	7.6e−11	8.5e−11	0.012	0.16
	E/W	<2e−16	<2e−16	<2e−16	<2e−16	9.7e−12	8.9e−12	0.0021	0.041
		Height (GIANT)							
	N/S	<2e−16	<2e−16	5.9e−5	0.16	<2e−16	<2e−16	2.5e−4	0.17
	E/W	<2e−16	<2e−16	1.4e−4	0.051	<2e−16	<2e−16	7.2e−5	0.014
		Weighted PS				Unweighted PS			

*P* value for non-linear association between component of birth location and polygenic score. For all models *n* = 321,439. Statistical adjustment was performed as follows: model 1: no adjustment; model 2: adjustment for genotyping array only; model 3: adjustment for genotyping array, 10 PCs and study participation centre; model 4: adjustment for genotyping array, 40 PCs and study participation centre

*N/S*  north/south axis of birth location, *E/W*   east/west axis of birth location, *PS* polygenic scores, *BMI* body mass index, *GIANT* Genetic Investigation of ANthropometric Traits, *EA* educational attainment, *SSGAC* Social Science Genetic Association Consortium

### Alignment of complex traits with geography

Having found evidence for association between genotypic variation and geography, we used general additive models to test for non-linear relationships between four exemplar complex traits and geography. Reported household income, measured BMI, reported age at completion of full-time education and reported number of siblings showed strong evidence for geographical stratification (*p* < 2e−16 for non-linear relationship between observed traits and axes of birth location).

### Co-incident latent structure produces biased estimates

**Fig. 4:**
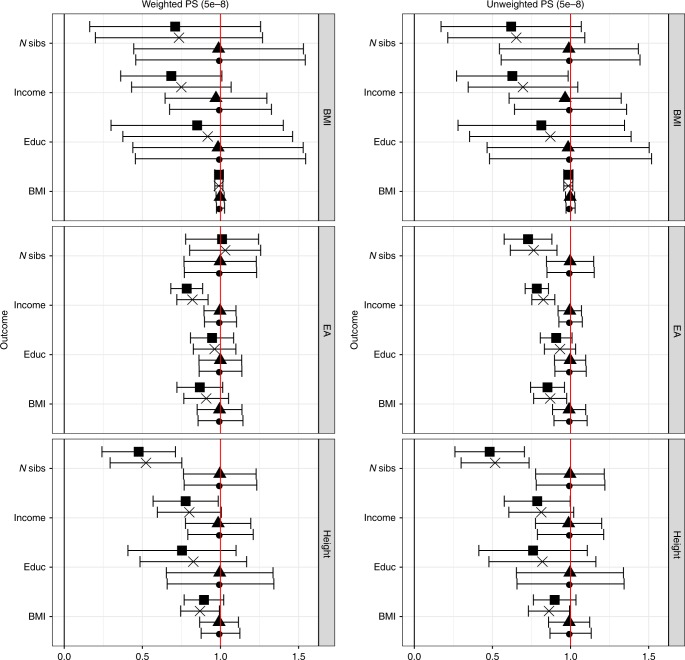
Attenuation in linear relationship between polygenic scores (PS) and complex traits in the UK Biobank sample at varying degrees of statistical adjustment. *N* sibs refers to number of siblings. For each PS, the relationship with four traits was estimated using an unadjusted model (plotted in circle) and this estimate and its corresponding 95% confidence intervals were rescaled to a value of 1. Error bars represent 95% confidence intervals for the rescaled estimate. Adjustment was then performed for genotyping array only (triangles), genotyping array, 40 principal components (PCs) and study participation centre (cross) and 40 PCs, study participation centre and non-linear regression terms for North and East axes of birth location (square). A value of 0.5 on the *y*-axis would mean that 50% of the unadjusted effect estimate remained after adjustment. Lines are drawn at *x* = 1 (red) and y = 0 (black) for reference

Given these observations, we hypothesised that latent structure might act as a source of covariance between genotypes and health outcomes, leading us to explore the potential role of latent structure in confounding analysis. We tested for linear association between the PS and complex traits and examined whether the inclusion of non-linear terms for birth location as covariates altered the results, again using general additive models. The relationship between the BMI PS and BMI changed little with increasing statistical adjustment, but other relationships changed in magnitude with the addition of non-linear terms for birth location. For example, the association between the BMI PS and household income attenuated by over 30% in a fully adjusted model compared to an unadjusted model, suggesting that the unadjusted estimate was confounded by co-incident latent structure in this sample. Similar patterns of attenuation were seen for both weighted and unweighted PS, and for strictly defined (Table 2, Fig. 4) and liberally defined PS (Supplementary Table 2 and Supplementary Fig. 2). Birth location captures neither the full extent of variation in fine ancestral structure (which predicts PS) nor the full extent of geographically structured social and economic differences (which predict income). It is possible that these adjusted estimates therefore contain residual confounding and that the true impact of biases within this sample is larger than these results suggest. Similarly, lack of association between a PS and birth location may be insufficient to assert that the PS is free from stratifying bias.

**Table. 2:** Linear relationships between observed traits and PS in UK Biobank

Observed trait (unit)	Model 1	Model 2	Model 3	Model 4	Model 1	Model 2	Model 3	Model 4
	PS for BMI (GIANT)							
Household income (£ per year)	−335 (1.8e−9)	−325 (5.2e−9)	−251 (4.0e−6)	−229 (3.4e−5)	−304 (4.7e−8)	−294 (1.3e−7)	−212 (1.0e−4)	−190 (0.0057)
BMI (kg/m^2^)	0.612 (<2e−16)	0.611 (<2e−16)	0.606 (<2e−16)	0.606 (<2e−16)	0.549 (<2e−16)	0.547 (<2e−16)	0.541 (<2e−16)	0.541 (<2e−16)
Age at completion of full-time education (years)	−0.0219 (3.2e−4)	−0.0216 (4.0e−4)	−0.0201 (9.2e−4)	−0.0187 (0.0025)	−0.0231 (1.6e−4)	−0.0227 (2.0e−4)	−0.0201 (9.6e−4)	−0.0187 (0.0024)
Number of siblings (persons)	0.0107 (3.0e−4)	0.0105 (3.6e−4)	0.00783 (0.0071)	0.00750 (0.011)	0.00130 (1.0e−05)	0.00129 (1.3e−05)	0.00850 (0.0035)	0.00807 (0.0068)
	PS for EA (SSGAC)							
Household income (£ per year)	1066 (<2e−16)	1062 (<2e−16)	874 (<2e−16)	835 (<2e−16)	1454 (<2e−16)	1446 (<2e−16)	1200 (<2e−16)	1140 (<2e−16)
BMI (kg/m^2^)	−0.112 (<2e−16)	−0.111 (<2e−16)	−0.101 (<2e−16)	−0.097 (<2e−16)	−0.151 (<2e−16)	−0.150 (<2e−16)	−0.132 (<2e−16)	−0.129 (<2e−16)
Age at completion of full-time education (years)	0.0878 (<2e−16)	0.0877 (<2e−16)	0.0844 (<2e−16)	0.0831 (<2e−16)	0.12 (<2e−16)	0.119 (<2e−16)	0.112 (<2e−16)	0.109 (<2e−16)
Number of siblings (persons)	−0.0250 (<2e−16)	−0.0250 (<2e−16)	−0.0258 (<2e−16)	−0.0253 (<2e−16)	−0.038 (<2e−16)	−0.0382 (<2e−16)	−0.0293 (<2e−16)	−0.0279 (<2e−16)
	PS for height (GIANT)							
Household income (£ per year)	522 (<2e−16)	515 (<2e−16)	418 (1.8e−14)	406 (2.7e−13)	515 (<2e−16)	509 (<2e−16)	419 (1.7e−14)	405 (2.9e−13)
BMI (kg/m^2^)	−0.129 (<2e−16)	−0.128 (<2e−16)	−0.112 (<2e−16)	−0.116 (<2e−16)	−0.122 (<2e−16)	−0.121 (<2e−16)	−0.105 (<2e−16)	−0.109 (<2e−16)
Age at completion of full-time education (years)	0.0350 (9.4e−9)	0.0348 (1.1e−8)	0.0289 (2.0e−06)	0.0263 (2.0e−05)	0.0349 (1.1e−08)	0.0347 (1.2e−08)	0.0286 (2.6e−6)	0.0265 (1.8e−5)
Number of siblings (persons)	−0.0249 (<2e−16)	−0.0248 (<2e−16)	−0.0130 (8.1e−06)	−0.0119 (7.2e−05)	−0.0264 (<2e−16)	−0.0263 (<2e−16)	−0.0136 (3.0e−6)	−0.0127 (2.1e−5)
	Weighted PS (*p* < 5e^−8^))				Unweighted PS (*p* < 5e^-8^)			

The field contents are beta coefficients per 1 SD increase in PS, with *p* values for the linear association, testing the null hypothesis of no linear association between each observed trait and PS in brackets. For household income, *N* = 276,779; BMI, *N* = 336,031; age at completion of full-time education, *N* = 228,886; number of siblings, *N* = 332,037. Statistical adjustment was performed as follows: model 1: no adjustment; model 2: adjustment for genotyping array only; model 3: adjustment for genotyping array, 40 PCs and study participation centre; model 4: adjustment for genotyping array, 40 PCs, study participation centre and non-linear regression terms for North and East axes of birth location

*PS* polygenic score, *PC*  principal component, *BMI*  body mass index, *EA*  educational attainment*, GIANT* Genetic Investigation of ANthropometric Traits, *SSGAC* Social Science Genetic Association Consortium

As an alternative way to demonstrate the potential impact of such bias, we generated random complex traits matched to real complex traits, assigning each participant a plausible value based on typical values for the corresponding real traits in their region of the UK Biobank sample. This procedure aimed to produce traits recapitulating the coarse geographical distribution of the real complex traits, while greatly reducing the magnitude of biological genotype–phenotype pathways, as the matched traits were uncorrelated with either the real genotypes or real traits within any given region (Methods). In this analysis, each PS was associated with at least one complex trait and these associations attenuated with adjustment for latent structure, collectively suggesting PS predict geographical location within the UK Biobank sample (Supplementary Table 3).

## Discussion

The presence of structure within the genetic data of UK Biobank has several potential explanations, including a legacy of ancient ancestral groups that are not fully admixed^18,35^, a consequence of non-random mating or polygenic selection^36–38^, a study artefact induced by selection bias^24^ or a combination of all these explanations. Regardless of origin, this phenomenon is important, both as a source of ecological-level covariance between genotypes and geographically heterogeneous complex traits, and because of its apparent persistence across different analytical contexts and modes of statistical adjustment. Recent evidence from an investigation in the United States^39^ also illustrates associations between PS and complex traits at the ecological level. Now manifest, this property should be added to the growing list of limitations to naive use of PS—including horizontal pleiotropy^12^, high false discovery rate^40^, association with coarse ancestral groups^41^ and prediction of inter-generational phenotypes which complicates interpretation^42^.

The ability of very large studies to detect effects indistinguishable from artefactual biases or ancestral differences demands reworked approaches to exploit^43^, or at least account for, structure. Exciting recent developments aim to improve statistical models^44^ or leverage information from family-based study designs for unbiased inference^45^. Until such methods have developed further, we hope this short article draws attention to an important phenomenon and illustrates the ongoing relevance of basic epidemiolocal principles in an era of increasingly sophisticated analyses.

## Methods

### ALSPAC

The ALSPAC is a birth cohort which recruited 14,541 pregnant women living in the former county of Avon (surrounding Bristol, UK) with expected delivery dates between 1 April 1991 and 31 December 1992. Since then, participating mothers and their children have been followed up with serial clinical data, questionnaire data and biosample collection. A nested cohort following children of the index offspring has been formed and data on fathers of the index offspring are also available. Further information on the index offspring and mothers cohorts are available^15,16^. The study website contains details of all data that are available through a fully searchable data dictionary at http://www.bris.ac.uk/alspac/researchers/data-access/data-dictionary/.

Ethical approval for the study was obtained from the ALSPAC Ethics and Law Committee and the Local Research Ethics Committees, and this research complied with all relevant ethical regulations. Participants gave informed consent. This publication is the work of the authors and N.T. and S.H. who will serve as guarantors for the contents of this paper.

### Genotypes

Genotype data for ALSPAC mothers were generated using the Illumina human660w genotyping array and genotypes were called with Illumina GenomeStudio, yielding 557,124 directly genotyped single-nucleotide polymorphisms (SNPs) in 10,015 participants. Participants and SNPs were carried through to imputation if they passed quality control measures implemented in PLINK (v1.07). SNP-level quality control removed variants with more than 5% missingness, or *p* value for Hardy–Weinberg equilibrium smaller than 1e−6. Participant-level quality control removed variants with uncertain X chromosome heterozygosity, extreme autosomal heterozygosity or more than 5% overall missingness. Next, multi-dimensional scaling of genome-wide data was performed including reference data from HapMap populations. Samples which clustered outside the CEU population were removed. Following these measures, data for 9048 participants and 526,688 SNPs were available. Related participants were identified by estimating inheritance by descent, where an estimate of greater than 0.125 was considered to represent cryptic relatedness. Data from these participants were included in phasing and imputation, but prior to analysis related individuals were removed from the dataset until no cryptically related pairs were present, yielding a final sample size of 8196 mothers.

### Chromosome painting

To infer within-UK ancestral origin of these mothers, we used Chromosome Painting^17^ coupled with high-resolution spatial data from the PoBI^18^. We first merged the ALSPAC mothers and PoBI data into a set of shared SNPs (using the ALSPAC imputed data). We then performed phasing using Shapeit^46^ and then Impute2^47^ to impute any remaining missing SNPs. Finally, we used ChromoPainter^17^ to paint each individual against all individuals from the 35 labelled populations given in PoBI to obtain a genome-wide estimate of haplotype sharing.

We then constructed a reference panel for the PoBI data by averaging the painting for all individuals in each of the labelled populations. We then used the Non-Negative Least Squares method for estimating ancestry^48^ for each of the ALSPAC participants, in terms of the 35 labels. In total, 7739 ALSPAC mothers and 2,039 PoBI participants were included in analyses.

We did not observe many individuals with ancestry from only a single PoBI region. Therefore, we have to infer the underlying population averages that, given the mixture that we observed, would have given rise to the data. We use the method of Lawson et al.^49^ for this. Let *AB*=*C*, where *A* is an *N* by *K* matrix of admixture estimates, *B* is the population phenotypes to be inferred, and *C* is the observed individual phenotype estimates, i.e., measured education. Then, we can solve *B*=(*A*^*T*^*A*)^−1^A^*T*^*C*.

We note that this procedure is solely used to generate a visualisation of the relationship between genetic information and migration status and is not intended to reflect inference regarding individual-level ancestry. At the individual-level, using genetically non-distinct populations (i.e. based on labels rather than genetic distinctness as was done in Leslie et al.^18^) could result in the inference being unidentifiable. For this question regarding average phenotype, this lack of identifiability does not matter since populations are represented in the correct proportions, on average. This is evidenced by the clear structure visible in Fig. 1.

### UK Biobank methods

The UK Biobank study assessment centre sites targeted densely populated areas of England, Scotland and Wales, where a large eligible population could attend in-person assessment with a journey of less than 10 miles^50^. Participants gave informed consent, and the UK Biobank was approved by the North West Multi-centre Research Ethics Committee. This research was conducted using the UK Biobank Resource applications 8786 and 15825, and complied with all relevant ethical regulations.

### Geographical data

The assessment centre at which a participant consented was assigned a numerical code (field 54 in the UK Biobank data). In analyses adjusted for assessment centre, these codes were treated as factor variables.

Participants who were born in the United Kingdom were asked to name their place of birth during a verbal interview at study assessment centres. These answers were used to derive approximate North and East co-ordinates (rounded values, recorded on a metre grid scale from an origin South-West of the UK, fields 129 and 130 in the UK Biobank data). Values less than zero were coded as missing for both variables.

### Complex traits

Household income was obtained from baseline data in UK Biobank. Participants were asked to report the annual income for their household in pounds sterling using one of the following options (1: less than 18,000; 2: 18,000 to 30,999; 3: 31,000 to 51,999; 4: 52,000 to 100,000; 5: greater than 100,000; −1: do not know; −3: prefer not to answer) (field 738). To allow inclusion in a linear regression model these categories were recoded to values in pounds sterling in the midpoint of each category. For categories with only an upper or lower bound, the difference between the midpoint and boundary of the next adjacent category was used to estimate a midpoint as follows (1: 11,505; 2: 22,495; 3: 41,499; 4: 76,000; 5: 124,000). Household income was coded as missing for participants who preferred not to answer or did not know their household income.

Number of siblings was obtained from baseline data. Participants were asked to report how many full brothers they had (including those who have died and twin brothers, but excluding half-brothers, step-brothers and adopted brothers) (field 1873). A matching question was asked for sisters (field 1883). The responses for these two questions were combined to create a number of full siblings. This variable was coded as missing for participants who preferred not to answer or did not know for either source question.

BMI was derived by UK Biobank from height and weight measures during the initial assessment centre visit (field 21001) as weight divided by height squared (units kg/m^2^). This variable was coded as missing if either height or weight measures were missing.

Age at completion of full-time education was taken from baseline data (field 845). Participants were asked to report their age (in years) when they completed full-time education. This variable was set to missing for participants who responded that they never went to school, did not know or preferred not to answer. This question was not asked for participants who had previously indicated they had a college or university degree and this is reflected in the smaller sample size compared to other complex traits.

### Randomly assigned traits

Participants were ranked by North/South axis of birth location within the United Kingdom, and divided into 100 bins, each with an equal number of participants. Within each bin, the mean and standard deviation of each complex trait (income, number of siblings, BMI and age at completion of full-time education) was summarised, then new values for that trait were drawn from a random distribution with the same mean and standard deviation. The procedure was repeated for East/West axis of birth location, yielding two new values for each simulated trait. These values were combined with equal weighting, producing a total of four simulated traits which aimed to preserve coarse geographical variation across the sampling frame of UK Biobank whilst greatly reducing or eliminating direct biological effects.

### Genetic data

We used the UK Biobank 500k (July 2017) genotype release, for which pre-imputation quality control, phasing and imputation are described elsewhere^20^. Following imputation we removed variants that were not present within the haplotype reference consortium (HRC) imputation panel and applied a graded filtering on imputation quality. Rarer variants were required to have a higher imputation INFO score (Info > 0.3 for minor allele frequency (MAF) > 3%; Info > 0.6 for MAF 1–3%; Info > 0.8 for MAF 0.5–1% and Info > 0.9 for MAF 0.1–0.5%). We removed 378 individuals with a mismatch between genetic sex and reported sex and 352 individuals with putative sex chromosome aneuploidy. We performed analysis within individuals who self-reported as “British” and had similar ancestral background from genetic PCs (*n* = 409,703). We applied an exclusion list containing 79,448 individuals, whilst preferentially removing individuals related to the greatest number of other individuals so that no related pairs remained in the final sample used for analysis. A comprehensive description of quality control methods has been published online^51^.

### Genetic principal components

We used genetic PCs supplied by UK Biobank (field 22009). These were calculated using a set of 407,219 unrelated, high-quality samples and 147,604 high confidence markers after pruning for linkage disequilibrium. Participants with missing PCs were excluded from analysis.

### Genome-wide association studies

GWAS for birth location were performed using PLINK (v2.0, August 2017 release)^25^. A full description of the analytical pipeline has been published online^52^. All models included adjustment for genotyping array and sex. Assessment centre was treated as a factor variable (where included) and PCs were treated as linear covariables.

### Polygenic scores

We took variants and weights associated with educational attainment from the discovery phase of a recent genome-wide meta-analysis (excluding the replication phase in UK Biobank)^28^. Variants and weights for height and BMI were taken from the entire meta-analysis results from the GIANT consortium^29,30^. For each of these three traits, we obtained clumped instruments from the MRBase repository using the “extract_instruments” option in the TwoSampleMR R package^53^. Effect allele dosage was extracted for these variants from the filtered UK Biobank genotype data. Effect allele dosage was weighted by reported genetic effect (beta) and then summarised across all contributing variants to create per-individual PS. Unweighted PS were created in parallel, which included the same variants but only considered direction of effect, not reported effect size. PS were *z*-transformed after application of all exclusion criteria. For sensitivity analysis, variants and weights for BMI were taken from published results in Biobank Japan^34^, clumped using reference data from East Asian ancestry participants in 1000 genomes and then used to derive PS following the same workflow as the main analysis.

### Generalised additive models

The relationship between complex traits and geographical parameters was modelled using the ‘mgcv’ package (version 1.8)^32^ in R (version 3.3.1)^33^. Traits were modelled against a spline function for either birth northings or birth eastings, in the form *t ~ s(location)*. Approximate statistical significance for non-linear terms was taken from the model summary, which estimates a suitable number of degrees of freedom from cross validation.

The relationship between PS and geographical parameters was modelled in a similar way, but incorporated a variable for genotyping array as the minimum adjustment, in the form *ps ~ s(location)* *+* *array*. Fully adjusted models included factor variables for study centre and up to 40 genetic PCs.

The relationship between complex traits and PS was modelled as a linear relationship to obtain indicative effect sizes, and took the form *t ~ ps*. Where relevant, non-linear covariables were included as spline terms in the form *t ~ ps* *+* *s(birth_location)* *+* *other covariables*. Simulated complex traits were modelled in exactly the same way as observed complex traits.

### Reporting summary

Further information on experimental design is available in the Nature Research Reporting Summary linked to this article.

## Supplementary information

Supplementary Information

Reporting Summary

## Data Availability

ALSPAC data are available through an access procedure described at http://www.bristol.ac.uk/alspac/researchers/access/. UK Biobank data are available through a procedure described at http://www.ukbiobank.ac.uk/using-the-resource/. POBI genotype data and location information are available via the European Genotype Archive (https://www.ebi.ac.uk/ega/) under accession numbers EGAS00001000672 and EGAD00010000632. Summary results of genome-wide association analysis are available at the University of Bristol data repository, data.bris, at 10.5523/bris.15pdhgrio8d6u2f2brgaahah9, and can also be queried using the LD-Hub (http://ldsc.broadinstitute.org/ldhub/) resource.
