# Supplementary material for: Apparent latent structure within the UK Biobank sample has implications for epidemiological analysis
Source: Nat Commun. 2019 Jan 18;10:333. doi: 10.1038/s41467-018-08219-1 (PMC6338768; doi:10.1038/s41467-018-08219-1)
Supplement: Supplementary file 1 — Supplementary Information [file 41467_2018_8219_MOESM1_ESM.pdf]

Supplementary Information for

Apparent latent structure within the UK Biobank sample has implications for epidemiological analysis

Haworth *et al*

Supplementary Figure 1: Quantile-Quantile plots of GWAS for birth location at varying degrees of statistical adjustment.

a

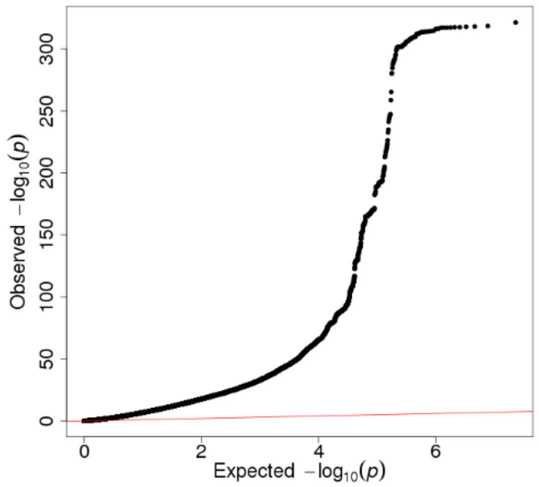

λ = 11.387

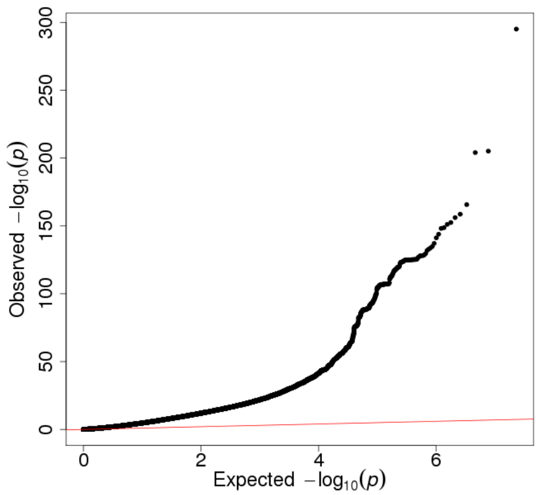

λ = 7.928

b

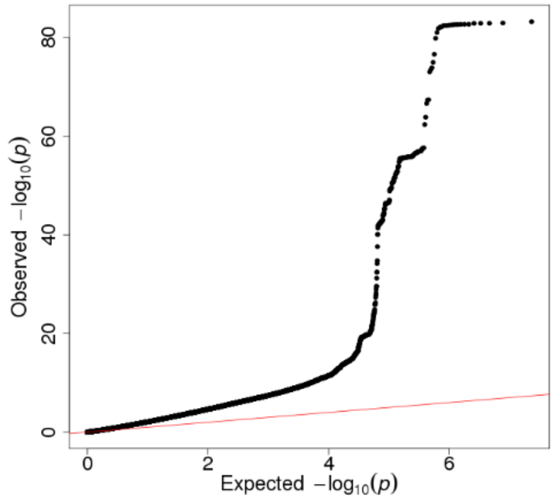

λ = 2.752

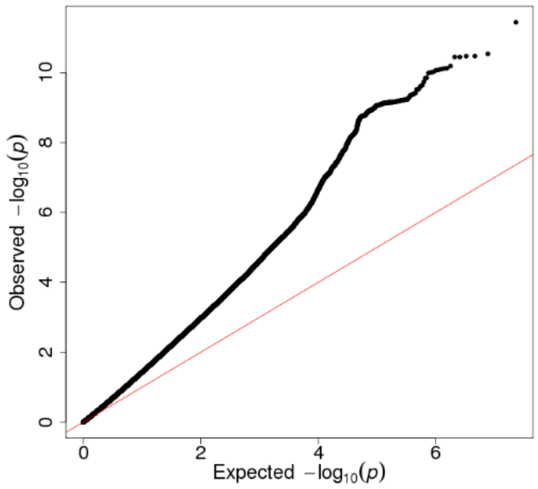

λ = 1.671

c

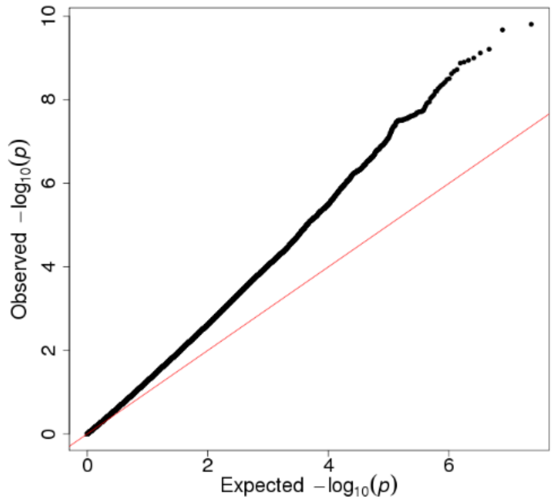

λ = 1.400

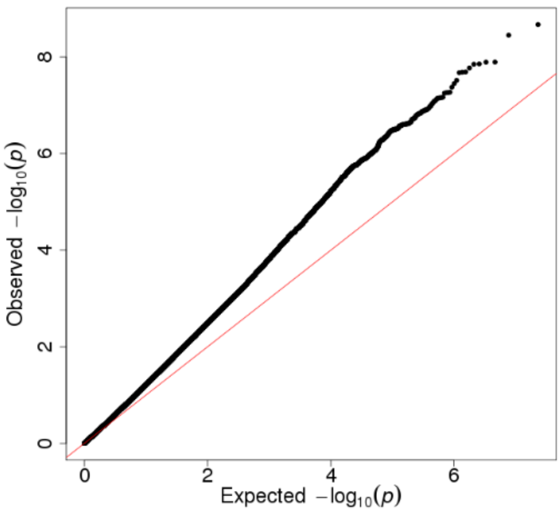

λ = 1.320

d

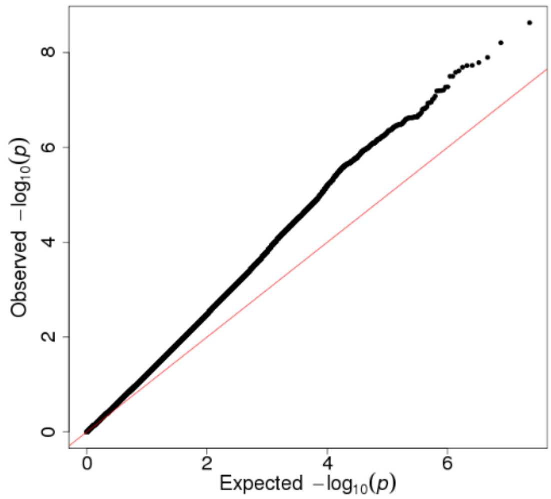

λ = 1.401

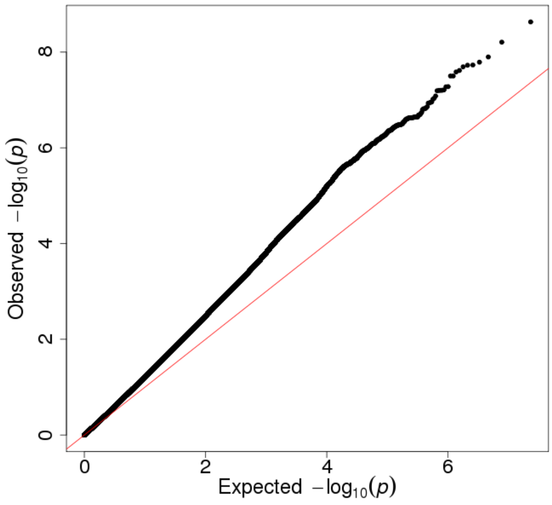

λ = 1.324

Plots on the left are for North/South axis of birth location, whilst those on the right are for East/West axis of birth location. 1a: Adjustment for genotyping chip and sex, 1b: Adjustment for genotyping chip, sex, 10 PCs, 1c: Adjustment for genotyping chip, sex, 40 PCs 1d, Adjustment for genotyping chip, sex, 40 PCs and assessment centre.

Supplementary Figure 2: Attenuation in linear relationships between PS and complex traits in the UK Biobank sample at varying degrees of statistical adjustment.

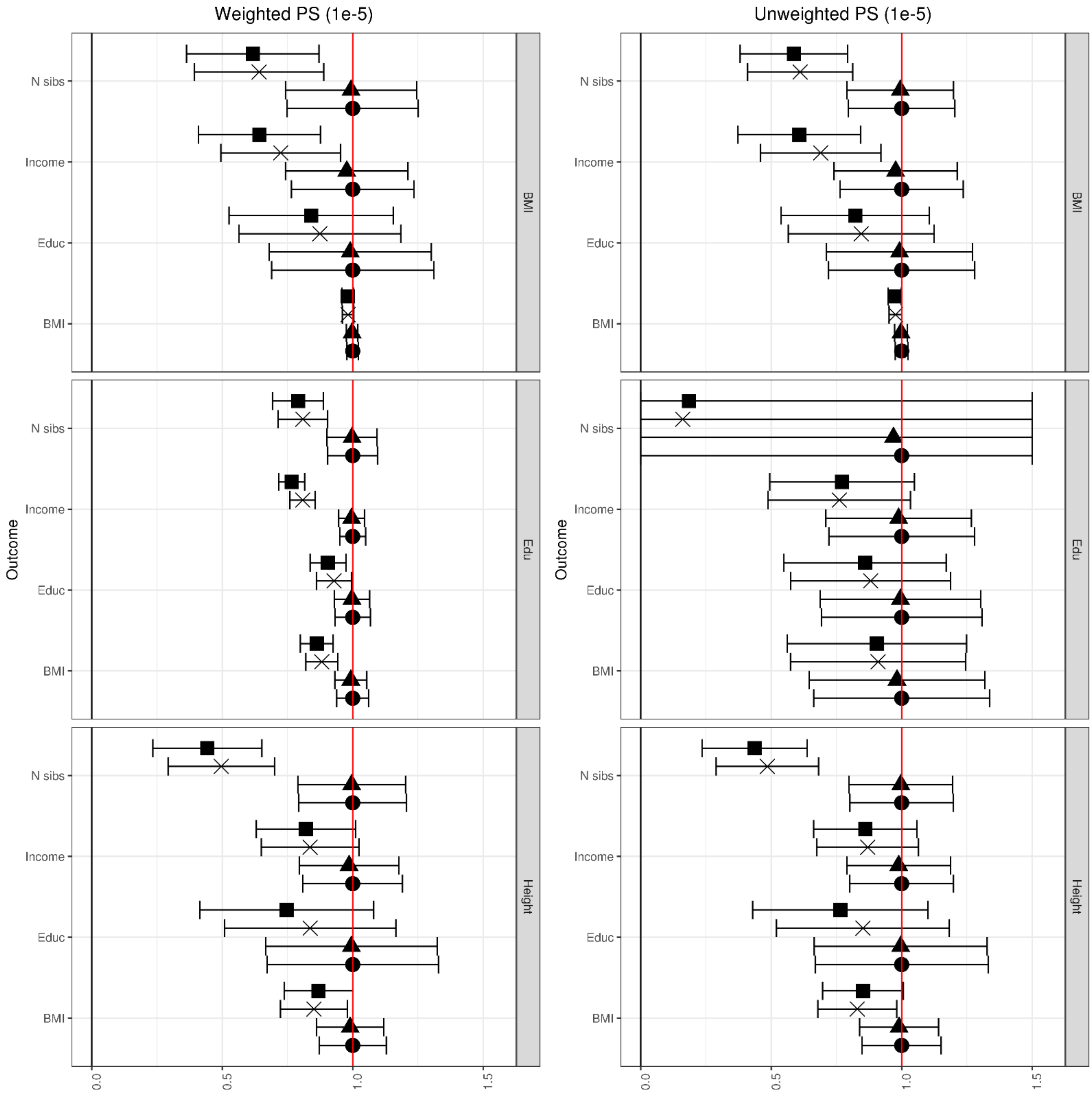

For each PS, the relationship with four traits was estimated using an unadjusted model (plotted in circle) and this estimate and it's corresponding 95% confidence intervals were rescaled to a value of 1. Adjustment was then performed for genotyping array only (triangles), genotyping array, 40 PCs and study participation centre (cross) and 40 PCs, study participation centre and non-linear regression terms for North and East axes of birth location (square). A value of 0.5 on the y-axis would mean that 50% of the unadjusted effect estimate remained after adjustment. Lines are drawn at x=1 (red) and x=0 (black) for reference. Error bars for the relationship between unweighted

educational attainment PS (1e-5) and number of siblings have been truncated at x=0 and x=1.5 to preserve the same scale across all comparisons.

Supplementary Table 1: Comparison of properties of polygenic scores for BMI trained in GIANT and Biobank Japan

		P value for association between PS and geographical term							
		Model 1	Model 2	Model 3	Model 4	Model 1	Model 2	Model 3	Model 4
P (5.0e-08)		BMI (GIANT)							
	North/South	9.7e-07	9.9e-07	0.063	0.40	0.0013	0.0012	0.0032	0.58
	East/West	0.0036	0.0035	0.24	0.93	0.053	0.054	0.032	0.47
		BMI (Biobank Japan)							
	North/South	6.0e-06	6.4e-06	0.14	0.096	3.2e-08	3.4e-08	0.11	0.088
	East/West	0.014	0.014	0.19	0.67	0.022	0.022	0.072	0.50
P (1.0e-05)		BMI (GIANT)							
	North/South	2.4e-09	2.5e-09	0.023	0.019	2.4e-10	2.6e-10	0.0029	0.074
	East/West	1.4e-13	1.7e-13	0.134	0.34	<2e-16	<2e-16	0.020	0.14
		BMI (Biobank Japan)							
	North/South	9.4e-11	9.9e-11	0.51	0.24	2.5e-04	2.5e-04	0.87	0.40
	East/West	8.7e-06	8.1e-06	0.059	0.039	0.025	0.024	0.17	0.098
		Weighted PS				Unweighted PS			

PS = Polygenic score, BMI = Body Mass Index. Table contents are p values for non-linear association with geographical axis. Statistical adjustment was performed as follows: model 1 – no adjustment; model 2 – adjustment for genotyping array only; model 3 – adjustment for genotyping array, 40 PCs and study participation centre; model 4 – adjustment for genotyping array, 40 PCs, study participation centre and spline regression terms for North and East axes of birth location.

Supplementary Table 2: Linear relationships between observed traits and PS in UK Biobank.

Observed trait (unit 1SD increase in PS $^{-1}$)	N	Model 1	Model 2	Model 3	Model 4	Model 1	Model 2	Model 3	Model 4
		PS for BMI (GIANT)							
Household income (£ year^{-1})	276,779	-465 (<2e-16)	-454 (3.5e-16)	-337 (6.4e-10)	-299 (7.1e-8)	-463 (<2e-16)	-452 (4.7e-16)	-319 (4.9e-9)	-281 (3.9e-7)
Body mass index (kg m^{-2})	336,031	0.72 (<2e-16)	0.72 (<2e-16)	0.71 (<2e-16)	0.71 (<2e-16)	0.65 (<2e-16)	0.65 (<2e-16)	0.63 (<2e-16)	-0.63 (<2e-16)
Age at completion of full time education (years)	228,886	-0.038 (2.9e-10)	-0.038 (4.2e-10)	-0.034 (3.2e-8)	-0.032 (1.6e-7)	-0.043 (2.6e-12)	-0.042 (3.9e-12)	-0.036 (3.1e-9)	-0.035 (1.3e-8)
Number of siblings (persons)	332,037	0.023 (6.0e-15)	0.023 (9.0e-15)	0.015 (4.0e-7)	0.014 (1.9e-6)	0.028 (<2e-16)	0.028 (<2e-16)	0.017 (2.7e-9)	0.017 (2.8e-8)
		PS for EA (SSGAC)							
Household income (£ year^{-1})	276,779	2201 (<2e-16)	2191 (<2e-16)	1779 (<2e-16)	1687 (<2e-16)	392 (2.2e-12)	387 (3.7e-12)	298 (3.5e-8)	302 (4.9e-8)
Body mass index (kg m^{-2})	336,031	-0.26 (<2e-16)	-0.26 (<2e-16)	-0.23 (<2e-16)	-0.23 (<2e-16)	-0.048 (6.1e-9)	-0.047 (1.1e-8)	-0.043 (1.1e-7)	-0.043 (2.4e-7)
Age at completion of full time education (years)	228,886	0.18 (<2e-16)	0.18 (<2e-16)	0.16 (<2e-16)	0.16 (<2e-16)	0.039 (1.9e-10)	0.039 (2.3e-10)	0.034 (1.8e-8)	0.033 (6.0e-8)
Number of siblings (persons)	332,037	-0.060 (<2e-16)	-0.060 (<2e-16)	-0.049 (<2e-16)	-0.048 (<2e-16)	-0.0024 (0.41)	-0.0023 (0.43)	-0.00039 (0.89)	-0.00045 (0.88)
		PS for height (GIANT)							
Household income (£ year^{-1})	276,779	573 (<2e-16)	565 (<2e-16)	479 (<2e-16)	470 (<2e-16)	550 (<2e-16)	544 (<2e-16)	479 (<2e-16)	474 (<2e-16)
Body mass index (kg m^{-2})	336,031	-0.12 (<2e-16)	-0.12 (<2e-16)	-0.11 (<2e-16)	-0.11 (<2e-16)	-0.11 (<2e-16)	-0.11 (<2e-16)	-0.089 (<2e-16)	-0.090 (<2e-16)
Age at completion of full time education (years)	228,886	0.036 (2.4e-9)	0.036 (2.9e-9)	0.030 (5.8e-7)	0.027 (1.1e-5)	0.036 (3.3e-9)	0.036 (3.8e-9)	0.031 (4.7e-7)	0.028 (8.2e-6)
Number of siblings (persons)	332,037	-0.028 (<2e-16)	-0.028 (<2e-16)	-0.014 (1.9e-6)	-0.012 (3.3e-5)	-0.029 (<2e-16)	-0.029 (<2e-16)	-0.014 (1.2e-6)	-0.013 (2.1e-5)
		Weighted PS (p<1e-5)				Unweighted PS (p<1e-5)			

PS = polygenic score; PC = principal component; SD = standard deviation; BMI = body mass index; EA = educational attainment. The field contents are beta coefficients per 1 SD increase in PS, with p-values for the linear association, testing the null hypothesis of no linear association between each observed trait and PS in brackets. Statistical adjustment was performed as follows: model 1 – no adjustment; model 2 – adjustment for genotyping array only; model 3 – adjustment for genotyping array, 40 PCs and study participation centre; model 4 – adjustment for genotyping array, 40 PCs, study participation centre and non-linear regression terms for North and East axes of birth location.

Supplementary Table 3: Linear relationships between random traits and polygenic scores

Simulated trait (unit for 1 SD increase in PS).	N	Model 1	Model 2	Model 3	Model 4	Model 1	Model 2	Model 3	Model 4
PS for BMI (GIANT)									
Household income (£ year^{-1})	276,779	-25.9 (0.0046)	-25.7 (0.00050)	-9.68 (0.093)	-6.46 (0.045)	-26.5 (0.00033)	-26.4 (0.00036)	-10.1 (0.081)	-7.32 (0.023)
Body mass index (kg/m-^{2})	336,031	0.00120 (0.0011)	0.00119 (0.0013)	0.000796 (0.0073)	0.000654 (0.0044)	0.00154 (2.9e-05)	0.00153 (3.5e-05)	0.000807 (0.0065)	0.000716 (0.0018)
Age at completion of full time education (years)	228,886	-0.00078 (0.12)	-0.00078 (0.11)	-0.00088 (0.029)	-0.00065 (0.028)	-0.00101 (0.043)	-0.00102 (0.042)	-0.000914 (0.024)	-0.000766 (0.010)
Number of siblings (persons)	332,037	0.00062 (0.0182)	0.00061 (0.0201)	4.86e-04 (0.017)	4.77e-04 (0.0030)	0.00091 (0.00060)	0.00090 (0.00069)	4.65e-04 (0.023)	4.77e-04 (0.0030)
PS for educational attainment (SSGAC)									
Household income (£ year^{-1})	276,779	100 (<2e-16)	100 (<2e-16)	52.6 (<2e-16)	20.4 (2.3e-10)	130 (<2e-16)	130 (<2e-16)	58.5 (<2e-16)	25.8 (<2e-16)
Body mass index (kg/m-^{2})	336,031	-0.00400 (<2e-16)	-0.00399 (<2e-16)	-0.00274 (<2e-16)	-0.00147 (1.6e-10)	-0.00689 (<2e-16)	-0.00688 (<2e-16)	-0.00291 (<2e-16)	-0.00178 (9.6e-15)
Age at completion of full time education (years)	228,886	0.00366 (3.0e-13)	0.00366 (3.0e-13)	0.00238 (4.8e-09)	0.00166 (2.9e-08)	0.00494 (<2e-16)	0.00495 (<2e-16)	0.00211 (2.1e-07)	0.00147 (9.0e-07)
Number of siblings (persons)	332,037	-0.00166 (3.6e-10)	-0.00165 (3.9e-10)	-0.00142 (3.4e-12)	-0.000606 (0.00017)	-0.00382 (<2e-16)	-0.00382 (<2e-16)	-0.00130 (1.8e-10)	-0.000694 (1.6e-05)
PS for height (GIANT)									
Household income (£ year^{-1})	276,779	64.7 (<2e-16)	64.6 (<2e-16)	23.4 (5.2e-05)	10.2 (0.0015)	65.4 (<2e-16)	65.3 (<2e-16)	23.6 (4.6e-05)	10.6 (0.0011)
Body mass index (kg/m-^{2})	336,031	-0.00507 (<2e-16)	-0.00506 (< 2e-16)	-0.000969 (0.0011)	-0.000664 (0.0039)	-0.00543 (<2e-16)	-0.00542 (<2e-16)	-0.00104 (0.00047)	-0.000737 (0.0014)
Age at completion of full time education (years)	228,886	0.00429 (< 2e-16)	0.00429 (< 2e-16)	0.00159 (8.5e-05)	0.000993 (0.00087)	0.00439 (<2e-16)	0.00439 (<2e-16)	0.00155 (0.00013)	0.000980 (0.0010)
Number of siblings (persons)	332,037	-0.00329 (< 2e-16)	-0.00328 (< 2e-16)	-8.67e-05 (0.67)	5.15e-05 (0.75)	-0.00363 (<2e-16)	-0.00362 (<2e-16)	-0.000163 (0.43)	-9.63e-06 (0.95)
		Weighted PS (p<5e-8)				Unweighted PS (p<5e-8)			

PS = polygenic score; PC = principal component; SD = standard deviation. The contents are beta coefficients per 1 SD increase in PS, with p-values for the linear association, testing the null hypothesis of no linear association between each simulated trait and PS, in brackets. Statistical adjustment was performed as follows: model 1 – no adjustment; model 2 – adjustment for genotyping array only; model 3 – adjustment for genotyping array, 40 PCs and study participation centre; model 4 – adjustment for genotyping array, 40 PCs, study participation centre and spline regression terms for North and East axes of birth location.
