# Supplementary material for: Apparent latent structure within the UK Biobank sample has implications for epidemiological analysis
Source: Nat Commun. 2019 Jan 18;10:333. doi: 10.1038/s41467-018-08219-1 (PMC6338768; doi:10.1038/s41467-018-08219-1)
Supplement: Supplementary file 2 — Reporting Summary [file 41467_2018_8219_MOESM2_ESM.pdf]

nature research

Corresponding author(s): Nicholas Timpson

Reporting Summary

Nature Research wishes to improve the reproducibility of the work that we publish. This form provides structure for consistency and transparency in reporting. For further information on Nature Research policies, see Authors & Referees and the Editorial Policy Checklist.

Statistical parameters

When statistical analyses are reported, confirm that the following items are present in the relevant location (e.g. figure legend, table legend, main text, or Methods section).

n/a	Confirmed	
☐	☒	The exact sample size (*n*) for each experimental group/condition, given as a discrete number and unit of measurement
☐	☒	An indication of whether measurements were taken from distinct samples or whether the same sample was measured repeatedly
☐	☒	The statistical test(s) used AND whether they are one- or two-sided<br>*Only common tests should be described solely by name; describe more complex techniques in the Methods section.*
☐	☒	A description of all covariates tested
☐	☒	A description of any assumptions or corrections, such as tests of normality and adjustment for multiple comparisons
☐	☒	A full description of the statistics including central tendency (e.g. means) or other basic estimates (e.g. regression coefficient) AND variation (e.g. standard deviation) or associated estimates of uncertainty (e.g. confidence intervals)
☐	☒	For null hypothesis testing, the test statistic (e.g. *F*, *t*, *r*) with confidence intervals, effect sizes, degrees of freedom and *P* value noted<br>*Give P values as exact values whenever suitable.*
☒	☐	For Bayesian analysis, information on the choice of priors and Markov chain Monte Carlo settings
☒	☐	For hierarchical and complex designs, identification of the appropriate level for tests and full reporting of outcomes
☒	☐	Estimates of effect sizes (e.g. Cohen's *d*, Pearson's *r*), indicating how they were calculated
☐	☒	Clearly defined error bars<br>*State explicitly what error bars represent (e.g. SD, SE, CI)*

Our web collection on statistics for biologists may be useful.

Software and code

Policy information about availability of computer code

Data collection	No new data were collected in this investigation.
Data analysis	Data analysis was undertaken using a range of freely available software tools as described in the methods. No custom software was used.

For manuscripts utilizing custom algorithms or software that are central to the research but not yet described in published literature, software must be made available to editors/reviewers upon request. We strongly encourage code deposition in a community repository (e.g. GitHub). See the Nature Research guidelines for submitting code & software for further information.

Data

Policy information about availability of data

All manuscripts must include a data availability statement. This statement should provide the following information, where applicable:

- Accession codes, unique identifiers, or web links for publicly available datasets
- A list of figures that have associated raw data
- A description of any restrictions on data availability

ALSPAC data are available through an access procedure described at [http://www.bristol.ac.uk/alspac/researchers/access/]. UK Biobank data are available through a procedure described at [http://www.ukbiobank.ac.uk/using-the-resource/]. POBI genotype data and location information are available via the European Genotype Archive [https://www.ebi.ac.uk/ega/] under accession numbers [EGAS00001000672] and [EGAD00010000632]. Summary results of genome-wide association

analysis are available at the University of Bristol data repository, data.bris, at [https://doi.org/10.5523/bris.15pdhgrio8d6u2f2brgaahah9], and can also be queried using the LD-Hub [(http://ldsc.broadinstitute.org/ldhub/]) resource.

Field-specific reporting

Please select the best fit for your research. If you are not sure, read the appropriate sections before making your selection.

☒ Life sciences ☐ Behavioural & social sciences ☐ Ecological, evolutionary & environmental sciences

For a reference copy of the document with all sections, see nature.com/authors/policies/ReportingSummary-flat.pdf

Life sciences study design

All studies must disclose on these points even when the disclosure is negative.

Sample size	No power calculations were performed, as there was no prior expectation on the extent of fine-scale structure in either ALSPAC or UK Biobank. The use of a sample size similar to those used in current analysis of complex traits is likely suitable - any fine scale structure too subtle to be visible here may not be contributing to observed results in other analyses of complex traits in UK Biobank
Data exclusions	Participants were excluded who had missing data on birth location, current location or the exemplar complex traits. As the focus was on fine-scale structure (rather than coarse ancestral origin), analysis was restricted to participants of white British ancestry. Standard exclusion criteria were applied to genetic data to ensure quality control, as described in the methods and referenced documentation.
Replication	Primary analyses in UK Biobank were conducted in a single dataset without external replication of findings. However, external sources of information were included where possible to explore findings further, for example using a polygenic score trained in Biobank Japan.
Randomization	There was no randomization. Instead, the focus of this article was to highlight that both genetic and phentoypic data in UK Biobank is likely non-randomly distributed at national scale
Blinding	No intervention was performed, so blinding was not required

Reporting for specific materials, systems and methods

Materials & experimental systems

n/a	Involved in the study
☒	☐ Unique biological materials
☒	☐ Antibodies
☒	☐ Eukaryotic cell lines
☒	☐ Palaeontology
☒	☐ Animals and other organisms
☐	☒ Human research participants

Methods

n/a	Involved in the study
☒	☐ ChIP-seq
☒	☐ Flow cytometry
☒	☐ MRI-based neuroimaging

Human research participants

Policy information about studies involving human research participants

Population characteristics	The ALSPAC mothers are women who were living in the Bristol area in the early 1990s, were pregnant and consented to take part in a birth cohort study. Further information is available in the ALSPAC mothers cohort profile paper included in the references. The UK Biobank participants are volunteer participants aged 40-69 years who consented to take part in a cohort study between 2006-2010. Further information is described in the Fry et al (2017) reference in the manuscript.
Recruitment	Participants were volunteers who agreed to participate in research studies. In the case of UK Biobank, less than 6% of the eligible population agreed to participate. This is described further in the Fry et al (2017) reference. As described in the discussion section of the manuscript, the sampling strategy may have contributed to apparent structure in genetic data, i.e the study may be structured even if the underlying population is not.
